# CpG Oligodeoxynucleotides Enhance the Efficacy of Adoptive Cell Transfer Using Tumor Infiltrating Lymphocytes by Modifying the Th1 Polarization and Local Infiltration of Th17 Cells

**DOI:** 10.1155/2010/410893

**Published:** 2010-10-20

**Authors:** Lin Xu, Chunhong Wang, Zhenke Wen, Ya Zhou, Zhongmin Liu, Yongjie Liang, Zengguang Xu, Tao Ren

**Affiliations:** ^1^Department of Immunology, Zunyi Medical College, Guizhou 563003, China; ^2^Department of Chest Medicine, Qingdao Chest Hospital, Shandong 266043, China; ^3^Institute for Immunobiology and Department of Immunology, Shanghai Medical College, Fudan University, Shanghai 200032, China; ^4^Department of Medical Physics, Zunyi Medical College, Guizhou 563003, China; ^5^Department of Cardiothoracic Surgery, East Hospital, Tongji Unversity School of Medicine, Shanghai 200120, China; ^6^Department of Respiratory Medicine, East Hospital, Tongji Unversity School of Medicine, 150 Jimo Road, Pudong New Area, Shanghai 200120, China; ^7^Department of Scientific Research, East Hospital, Tongji Unversity School of Medicine, Shanghai 200120, China

## Abstract

Adoptive cell transfer immunotherapy using tumor infiltrating lymphocytes (TILs) was an important therapeutic strategy against tumors. But the efficacy remains limited and development of new strategies is urgent. Recent evidence suggested that CpG-ODNs might be a potent candidate for tumor immunotherapy. Here we firstly reported that CpG-ODNs could significantly enhance the antitumor efficacy of adoptively transferred TILs in vivo accompanied by enhanced activity capacity and proliferation of CD8^+^ T cells and CD8^+^ T cells, as well as a Th1 polarization immune response. Most importantly, we found that CpG-ODNs could significantly elevate the infiltration of Th17 cells in tumor mass, which contributed to anti-tumor efficacy of TILs in vivo. Our findings suggested that CpG ODNs could enhance the anti-tumor efficacy of adoptively transferred TILs through modifying Th1 polarization and local infiltration of Th17 cells, which might provide a clue for developing a new strategy for ACT based on TILs.

## 1. Introduction

In the past decades, adoptive cell transfer (ACT) immunotherapy has developed into an important therapeutic strategy against tumors. Many attempts have been made to improve the efficacy of adoptively transferred cells [1]. It was reported that adoptively transfer of tumor-specific T cell receptors-engineered T cells clone could improve the effect of immunotherapy [2]. Other researchers further adoptively transferred chimeric antigen receptor-engineered T cells into tumor host for immunotherapy of cancer [3]. However, the efficacy of ACT was still limited. Recent evidence suggested that tumor infiltrating lymphocytes (TILs) were the best candidate for ACT because of their directed interaction with tumor cells [4, 5]. However, the efficacy of TILs-based ACT was also intractable [6, 7]. Thus, new strategies were required for achieving effective antitumor responses of ACT based on TILs, which might ultimately aid the clinical therapy for tumor patients. 

CpG dinucleotides (CpG-ODNs) were strong activators of both innate and adaptive immunity through activating TLR9 molecule expressed on immunological cells such as dendritic cells, macrophages, T cells, and B cells. Over the past years, there has been an enormous increase in the understanding of the molecular and cellular effects of CpG-ODNs, which have been found to function as Th-1 adjuvant. This led to the idea to utilize CpG-ODNs for induction of antitumor immune response as an adjuvant therapeutic strategy [8, 9]. Our previous study found that CpG-ODNs could enhance the antitumor responses of peripheral blood mononuclear cells (PBMCs) from human lung cancer patients [10]. However, the effects of CpG-ODNs on ACT based on TILs remain unclear, which might be useful for optimizing the antitumor efficiency of adoptively transferred TILs and the development of new strategies for ACT immunotherapy. 

To address this issue, here we carefully evaluated the effect of CpG-ODNs on the antitumor efficacy of transferred TILs from human lung cancer patients. We demonstrated that CpG-ODNs could effectively enhance the antitumor responses of TILs through elevating activity capacity and proliferation of CD4^+^ and CD8^+^ T cells*.* Most importantly, we found that CpG-ODNs could modify the Th1 polarization and infiltration of Th17 cells in tumor mass. Our findings suggested that CpG-ODNs could enhance the antitumor responses of TILs, which could lead to a new understanding of the role of CpG-ODNs on ACT to enhance the antitumor efficacy.

## 2. Materials and Methods

### 2.1. Patients

Between Jan 2007 and Sep 2009, we collected tumor samples from patients with lung cancer in East Hospital, Shanghai, China. The study group (*n* = 26) comprised chemotherapy and radiotherapy naive patients with lung cancer. All patients gave informed consent approved by the local Ethics Committee. Review of pathology reports confirmed the diagnosis. Information regarding clinical pathological characters of patients was summarized in Table 1.

### 2.2. Isolation of TILs

The lymphocytes were harvested from tumors by a discontinuous density gradient method as described previously [11]. Briefly, tumors were removed aseptically and minced with scissors into 1-2 mm^3^ pieces. The minced tumors were then stirred in 40 mL complete RPMI 1640 containing 40 mg collagenase, type IV (Sigma), 4 mg deoxyribonuclease (Sigma), and 100 U hyaluronidase (Sigma) for 3 hours at room temperature. The tumor cell suspension was filtered through a nylon-mesh screen with pores of 50 *μ*m to remove cell clumps, and the filtrate was then centrifuged (250 g, 10 minutes). The cell pellet was washed and resuspended in complete RPMI 1640 (Life Technologies, Gaithersburg, MD). A 4-mL aliquot of cell suspension of disaggregated tumor was placed on top of the gradient formed by overlapping a cushion of 100% Ficoll-Paque (Pharmacia Fine Chemicals, Piscataway, NJ) with an equal volume of 75% Ficoll-Paque in RPMI 1640. Gradients (14 mL) were centrifuged at 800 g for 30 minutes at room temperature. The cells were collected and washed three times in fresh medium and resuspended in complete RPMI 1640 for future use.

### 2.3. Reagents

The following ODNs were used and purchased from Integrated DNA Technologies (Coralville, IO): CpG ODN (ODN 2006) 5′-TCGTCGTTTTGTCGTTTTGTCGT T-3′; control, (ODN1612) GCTAGAGCTTAGGCT. CpG ODN has a phosphorothioate backbone that provides a high degree of nuclease resistance. All the other reagents were purchased from Sigma-Aldrich unless stated otherwise.

### 2.4. Cell Preparations

 The autologous tumor cells from human lung cancer patients were prepared as previously [10]. Cells were cultured in RPMI 1640, supplemented with 10% fetal bovine serum (FBS), penicillin/streptomycin, and L-glutamine (all reagents purchased from Gibco-BRL, Grand Island, NY).

### 2.5. Adoptive Cell Transfer

 After stimulation with 10 ug/mL CpG-ODNs or control CpG-ODNs for 48 hours in the presence of 50 U/mL rIL-2, TILs were collected respectively. 1 × 10^7^ cells/mouse TILs in a 0.2-mL volume were injected intravenously into tumor-bearing nude mice with corresponding 100 mg CpG ODNs or control CpG ODNs, respectively. Tumor growth were measured at indicated times. Subsequent to adoptive transfer in vivo, bulk lymphocytes were isolated from tumors at the indicated time points.

### 2.6. Evaluation of Tumor Growth

Evaluation of tumor growth was performed as described previously with minor modifications [10]. Briefly, BALB/c nu/nu mice (6–8 weeks old) were injected subcutaneously with 0.2 mL of a single-cell suspension containing 5 × 10^5^ tumor cells and kept in laminar flow cabinets under specific pathogen-free conditions. After 7 days, about 70% mice developed tumor and the mean of tumor size was 6 mm^2^. Then 1 × 10^7^ autologous TILs pretreated as above described in a 0.2-mL volume were injected intravenously into tumor-bearing nude mice with corresponding CpG ODNs or control CpG ODNs respectively for the following experiments.

### 2.7. Flow Cytometry

 Flow cytometry was performed on an FACS Calibur (BD Biosciences) with CellQuest Pro software using directly conjugated mAbs against the following markers: CD3-FITC, CD4-PerCP, CD8-allophycocyanin, CD62L-PE, CD44-PE, IL-4-PE, or IFN-*γ*-PE with corresponding isotype-matched controls (either BD Biosciences or eBioscience Systems). Intracellular staining was run according to the manufacturer's protocol. To determine the percentage of CD4^+^Th17 cells, lymphocytes were gated by plotting forward versus side scatter followed by gating on CD3^+^ CD4^+^ T cells, and these cells were then analyzed for IL-17 expression.

### 2.8. In Vivo BrdU Labeling Proliferation

 Four days after adoptive cell transfer, tumor-bearing nude mice were administrated with 2 mg BrdU (5-bromo-2-deoxyuridine, Sigma) i.p. every other day up to a cumulative dose of 8 mg BrdU as indicated. Eight hours after the last BrdU injection, CD4^+^ T cells and CD8^+^ T cells isolated from the tumor mass were analyzed by flow cytometry for the incorporation of BrdU as described previously [12].

### 2.9. Detecting the Activated Phenotypes on Lymphocytes

 Ten days after adoptive cell transfer, tumors were sectioned and cut into pieces and then suspended in RPMI1640 containing 1 mg/mL collagenase IV for 4 hours. Lymphocytes infiltrated in the tumors were isolated by loading onto Ficoll for density gradient centrifugation. TILs were then directly conjugated with mAbs marked as CD3-FITC, CD4-PerCP, CD8-Allophycocyanin, CD62L-PE, or CD44-PE. Flow cytometry was performed on an FACS Calibur (BD Biosciences) with CellQuest Pro software. To determine the activated phenotype of T cells, lymphocytes were gated by plotting forward versus side scatter followed by gating on CD3^+^CD4^+^ T cells or CD3^+^ CD8^+^ T cells. Gated cells were then analyzed for CD44 and CD62L expression. The expression level of CD44 and CD62L on lymphocytes analyzed by FACS was shown as mean fluorescence intensity (MFI).

### 2.10. Intracellular Staining for IFN-*γ* and IL-4

TILs were harvested from tumor bearing nude mice and stained of surface markers (CD8, CD4); cells were fixed and permeabilized using Cytofix/Cytoperm and Perm/Wash buffer from BD Biosciences according to the manufacturer's instructions. All antibodies to cytokines (IFN-*γ*  and IL-4) including the corresponding isotype controls were obtained from BD Biosciences. Cells were stained with antibody against IFN-*γ*  or IL-4 (1 : 100) at 4°C for 30 minutes and washed twice in Perm/Wash before analysis.

### 2.11. Statistical Analyses

Statistical analyses of the data were performed with the SPSS12.0 software. Data were analyzed using a one-way analysis of variance (ANOVA) or Kruskal-Wallis test with PRISM 4.0 (GraphPad Soft-ware Inc, San Diego, CA, USA). *P < .05 was considered statistically significant in all comparisons.

## 3. Results

### 3.1. CpG-ODNs Treated TILs Reduced Tumor Burden and Prolonged Nude Mice Survival

**Figure 1:**
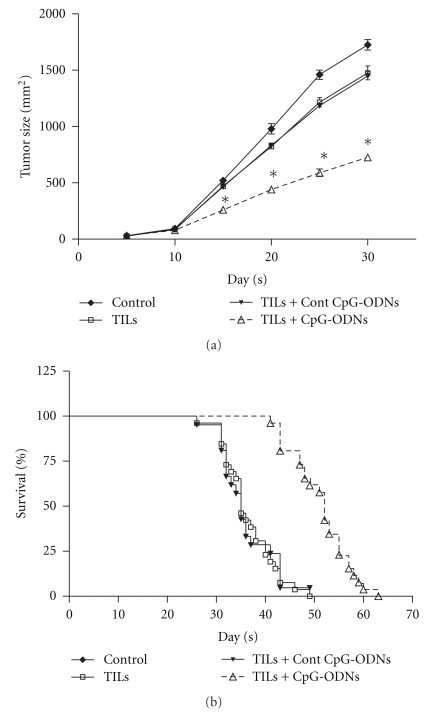
CpG-ODNs treated TILs reduced tumor burden and prolonged nude mice survival. TILs were collected from lung cancer patients (*n* = 26) and treated with CpG-ODNs or control CpG-ODNs as described in *Materials and Methods*. 1 × 10^7^ autologous TILs were transferred into lung cancer bearing Balb/c nude mice with 100 ug CpG-ODNs or control CpG-ODNs through tail vein. The tumor growth of each group was measured and the survival time of tumor bearing nude mice was calculated. One representative data from 26 individual patients with lung cancer was shown (a). Each bar represents the means (±SD) from 6 nude mice in each group. The survival rate from all 26 individual patients was also calculated (b). *P < .05.

 In order to evaluate the effect of CpG-ODNs on the antitumor efficacy of ACT immunotherapy, TILs isolated from human lung cancer patients were adoptively transferred into autologous tumor bearing nude mice with CpG-ODNs as described in Materials and Methods. Then the tumor growth and survival of tumor-bearing mice were observed. As shown in Figures 1(a) and 1(b), compared with control group, transferred autologous TILs significantly reduced the growth of tumor and prolonged the survival of tumor-bearing mice (*P* < .05). Importantly, we found that the tumor growth was dramatically reduced in the CpG-ODNs treated group compared with that of the TILs transferred group and Control CpG-ODNs treated group (Figure 1(a)). Moreover, the survival of tumor-bearing mice was significantly prolonged (Figure 1(b), day 53 versus days 34 and 36, *P* < .05). These results suggested that CpG-ODNs could enhance the antitumor efficiency of adoptively transferred TILs. 

### 3.2. CpG-ODNs Enhanced the Activation Capacity of Adoptively Transferred TILs

**Figure 2:**
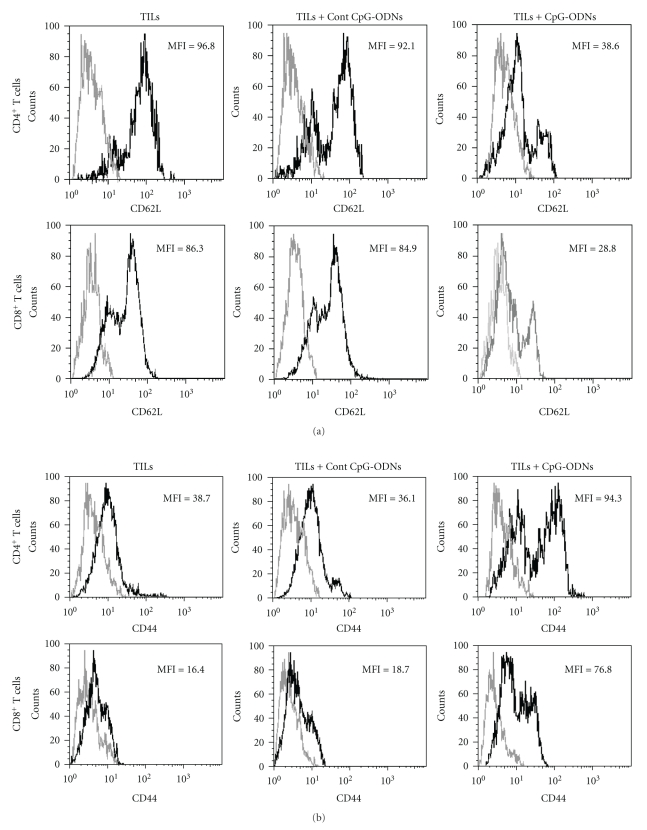
CpG-ODNs altered the expression of CD62L and CD44 on CD4^+^ T cells and CD8^+^ T cells. TILs were collected from lung cancer patients (*n* = 16) and treated with CpG-ODNs or control CpG-ODNs as described in *Materials and Methods*. 1 × 10^7^ autologous TILs were transferred into the tumor bearing Balb/c nude mice with 100 ug CpG-ODNs or control CpG-ODNs through tail vein. Ten days later, the lymphocytes were isolated from tumor mass and the expression of CD62L (a) and CD44 (b) on CD4^+^ T cells and CD8^+^ T cells was analyzed by FACS. Representative data referring to the mean of data obtained from all the patients included in each group was shown. *P < .05.

Then, we investigated whether the adoptively transferred TILs possessed the capacity for activation, which could contribute to antitumor effects of TILs *in vivo*. Ten days after transfer, the CD4^+^ T cells and CD8^+^ T cells were isolated from tumor mass and analyzed for their expression of surface activation marker CD62L and CD44. As shown in Figure 2(a), both CD4^+^ and CD8^+^ T cells in the CpG-ODNs treated group expressed lower MFI (mean fluorescence intensity) of CD62L which represented higher activation compared to the controls. In contrast, the expression of CD44 was significantly higher in the CpG-ODNs treated group (Figure 2(b), *P* < .05). These data suggested that the CpG-ODNs treated T cells possessed activation capacity and displayed an effector-memory like phenotype. 

### 3.3. CpG-ODNs Enhanced the Proliferation Capacity of Transferred TILs

**Figure 3:**
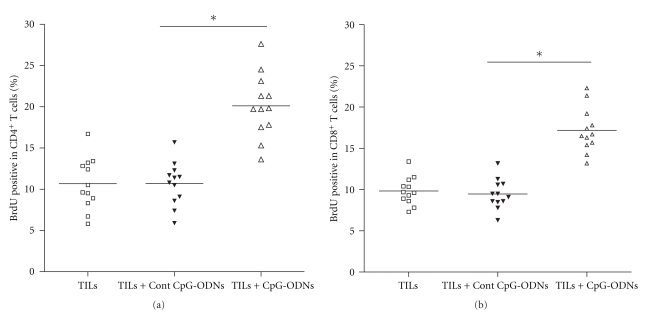
CpG-ODNs enhanced the local proliferation of CD4^+^ T cells and CD8^+^ T cells. TILs were collected from lung cancer patients (*n* = 12) and treated with CpG-ODNs or control CpG-ODNs as described in Materials and Methods. 1 × 10^7^ autologous TILs were transferred into the tumor bearing Balb/c nude mice with 100 ug CpG-ODNs or control CpG-ODNs through tail vein. Four days later, the recipients were treated with 2 mg BrdU i.p. every other day and up to a cumulative dose of 8 mg BrdU. Eight hours after the last BrdU injection, the lymphocytes were isolated from tumor mass and the proliferation of CD4^+^ T cells (a) and CD8^+^ T cells (b) was examined by FACS. *P < .05.

Next, we evaluated the proliferation capacity of adoptively transferred TILs. Four days after transfer, the tumor bearing nude mice were administrated with 2 mg BrdU every other day and up to a cumulative dose of 8 mg BrdU. Eight hours after the last BrdU injection, CD4^+^ T cells and CD8^+^ T cells were isolated from the tumor mass and analyzed for the incorporation of BrdU. Results shown that the proportion of BrdU^+^ in CD4^+^ T cells in CpG-ODNs treated group was higher than that in control groups (Figure 3(a), 21.4% versus 11.3% and 11.6%, *P* < .05). Moreover, the proportion of BrdU^+^ in CD8^+^ T cells in CpG-ODNs treated group also was higher than that in control groups (Figure 3(b), 16.5% versus 10.1% and 9.7%, *P* < .05). These data suggested that CpG-ODNs could enhance the proliferation of tumor infiltrating CD4^+^ T cells and CD8^+^ T cells. 

### 3.4. CpG-ODNs Promoted Polarization towards Th1 Immune Response of Transferred TILs

**Figure 4:**
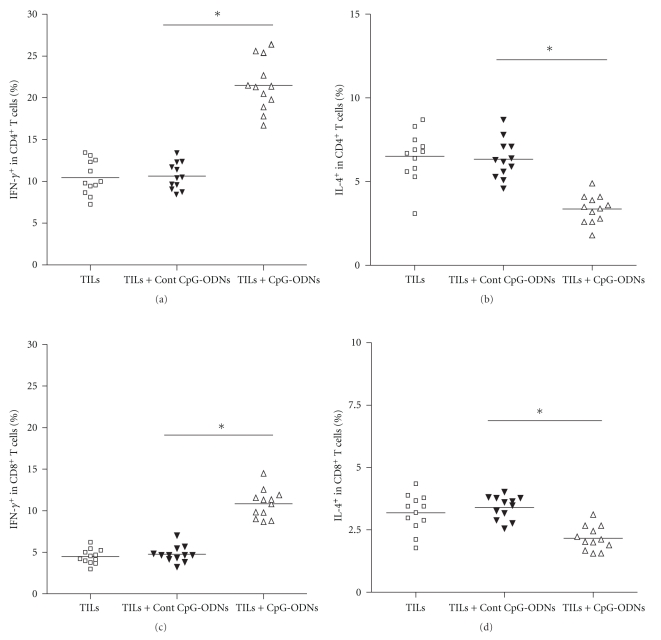
CpG-ODNs enhanced the IFN-*γ* production of CD4^+^ T cells and CD8^+^ T cells. TILs were collected from lung cancer patients (*n* = 12) and treated with CpG-ODNs or control CpG-ODNs as described in Materials and Methods. 1 × 10^7^ autologous TILs were transferred into the tumor bearing Balb/c nude mice with 100 ug CpG-ODNs or control CpG-ODNs through tail vein. Ten days later, CD4^+^ T cells and CD8^+^ T cells were isolated from tumor mass and their production of IFN-*γ* (a and c) and IL-4 (b and d) was detected by intracellular staining. *P < .05.

To gain an insight into whether the adoptively transferred TILs could induce Th1 immune response which dominated antitumor immunity, ten days after transfer, the CD4^+^ T cells and CD8^+^ T cells were isolated from the tumor mass and analyzed for their production of IFN-*γ* and IL-4. Results showed that the production of IFN-*γ* in CD4^+^ T cells and CD8^+^ T cells was significantly higher in the CpG-ODNs treated group compared with that of the control groups (Figures 4(a) and 4(c), *P* < .05). Reversely, the production of IL-4 in CD4^+^ T cells and CD8^+^ T cells was significantly lower in the CpG-ODNs treated group (Figures 4(b) and 4(d), *P* < .05). These data suggested that adoptive transfer of CpG-ODNs treated TILs skewed the immune response towards Th1, which greatly improved antitumor efficacy. 

### 3.5. CpG-ODNs Modified the Infiltration of Th17 Cells in Tumor Mass

**Figure 5:**
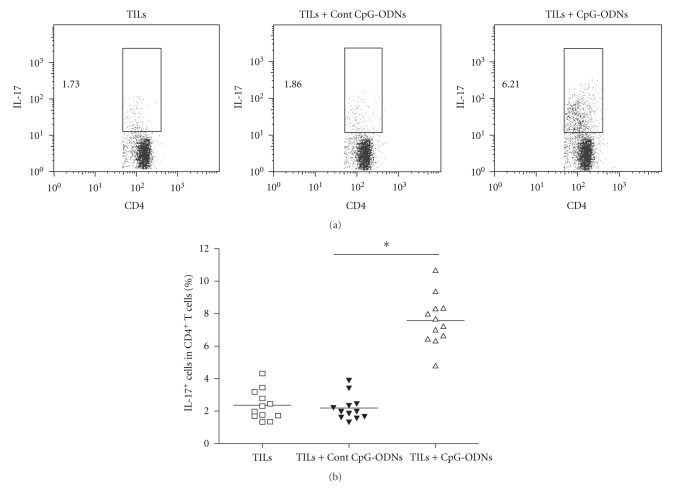
CpG-ODNs enhanced the infiltration of Th17 cells in tumor mass. TILs were collected from lung cancer patients (*n* = 12) and treated with CpG-ODNs or control CpG-ODNs as described in *Materials and Methods*. 1 × 10^7^ autologous TILs were transferred into the tumor bearing Balb/c nude mice with 100 ug CpG-ODNs or control CpG-ODNs through tail vein. Ten days later, the percentages of IL-17 positive cells in CD4^+^ T cells isolated from tumor mass were detected by flow cytometry. Representative data from 12 individual patients with lung cancer was shown (a), and the mean frequency of Th17 cells also was calculated, respectively (b). *P < .05.

Accumulating data suggested that Th17 cells played a pivotal role in antitumor immunity [13–15]. To assess the potential role of CpG-ODNs on Th17 cells in adoptive transferred TILs, ten days after transfer, T cells were isolated from the tumor mass and analyzed for the proportion of Th17 cells using flow cytometry. We found that the proportion of Th17 cells in CD4^+^ T cells was significantly higher in the CpG-ODNs treated group compared with that of the control groups (Figure 5(b), 7.64% versus 2.17% and 2.06%, *P* < .05). This result indicated that CpG-ODNs could modify the infiltration of Th17 cells in tumor mass in vivo. 

### 3.6. Inhibition of the Functional Activity of Th17 Cells Abrogated CpG-ODNs Enhanced Antitumor Efficiency of Transferred TILs

**Figure 6:**
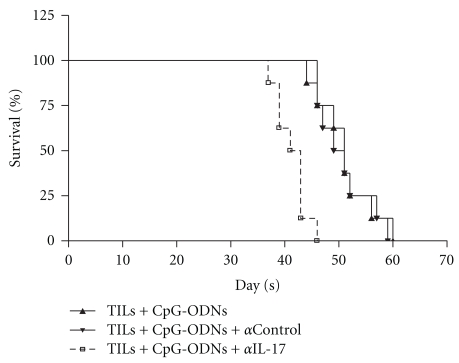
Neutralization of IL-17 reduced the survival of human lung cancer bearing nude mice. TILs were collected from lung cancer patients (*n* = 12) and treated with CpG-ODNs or control CpG-ODNs as described in *Materials and Methods*. 1 × 10^7^ autologous TILs were transferred into the tumor bearing Balb/c nude mice with 100 ug CpG-ODNs or control CpG-ODNs through tail vein. After 2 days, the recipients were injected intravenously with anti-IL-17 (5 ug/g) every seven days. Then, the survival of tumor bearing nude mice was observed.

To evaluate the potential role of Th17 cells in the enhanced antitumor responses of TILs induced by CpG-ODNs, two days after transfer, the recipients were injected intravenously with anti-IL-17 (5 ug/g) every seven days, and then the survival time of tumor-bearing mice were observed. Compared with that of control groups, the survival time in mice treated with IL-17 neutralizing antibodies was significantly reduced (Figure 6, day 40 versus days 48 and 49, *P* < .05), indicating that Th17 cells might play an important role in the enhanced antitumor efficacy of TILs induced by CpG-ODNs. 

## 4. Discussion

In the present study, we reported that CpG-ODNs could enhance the antitumor efficacy of adoptive transfer of TILs into tumor-bearing nude mice which was accompanied by increased activation and proliferation in both CD4^+^ and CD8^+^ T cells, as well as the polarization of Th1 immune response. More importantly, we found that CpG-ODNs could modify the infiltration of Th17 cells in tumor mass, which might contribute to the efficacy of ACT. 

Recently, CpG-ODNs were used as adjuvant in therapy against infections and cancer [16, 17]. However, the direct effects of CpG-ODNs on efficiency of transferred TILs remain unclear. It was reported that CpG-ODNs could elevate the activity capacity of T cells in tumor mass [18, 19]. We extended previous finding by demonstrating that the CpG-ODNs could enhance the antitumor efficacy of adoptive transferred TILs, which was correlated to enhanced activity and proliferation of tumor infiltrating CD4^+^ T cells and CD8^+^ T cells. It was consistent with our previous observation that CpG-ODNs could enhance the antitumor effects of PBMCs [10]. In this study, we further reported that CpG-ODNs could induce the Th1-type immune response in TILs adoptively transferred recipients. These results were consistent with other's work which showed that CpG-ODNs could stimulate the IFN-*γ* secretion of T cells and DCs in tumor host [20]. 

Recent study suggested that Th17 cells, a distinct subset of CD4^+^ T cells, infiltrated in tumor mass and played a controversial role in tumor immunity [14, 15]. Some research works reported that IL-17A, IL-23, and IL-6 could promote tumor growth and/or impair the function of effector T cells, suggesting that Th17 cells might play a negative role in antitumor immunity [21–23], while others showed that Th17 cells and Th-17 cell-associated cytokines could elevate antitumor immunity in some certain animal models [15, 24, 25]. Recent evidence further showed that adoptive transferred Th17-polarized cells could reduce the tumor growth in vivo [26]. Perhaps it reflected the fact that Th17 cells might play distinct roles in antitumor immune responses depending on the certain context of the experimental conditions [27–30]. In our study, we found that CpG-ODNs could elevate the infiltration of Th17 in tumor mass. Most importantly, neutralization of biological activity of IL-17 could significantly reduce CpG-ODNs enhanced efficiency of adoptive transferred TILs, suggesting that Th17 contributed to the CpG-ODNs enhanced antitumor immunity of adoptive transferred TILs. Consistently, some researches found that endogenous IL-17 contributed to reduced tumor growth and metastasis in vivo [24]. In contrast, Muranski et al. reported that not IL-17 but IFN-*γ* produced by Th17 cells contributed to tumor reduction [26]. We proposed that the possible role of Th17-derived cytokines such as IL-17 and IFN-*γ* in Th17 cell-mediated antitumor immunity might be dependent on their local concentrations, bioavailability, and potential targets [31]. However, the exact mechanism through which Th17 contributed to CpG-ODNs enhanced antitumor immunity of ACT remains as a subject to successive researches. 

In conclusion, our study demonstrated that CpG-ODNs could enhance the efficiency of adoptive transfer immunotherapy using TILs *in vivo* via modifying the Th1 polarization and local infiltration of Th17 cells, which might provide a useful strategic alternative for clinical biotherapy.

## 5. Conclusions

CpG-ODNs could enhance the efficiency of adoptive cell transfer immunotherapy based on tumor infiltrating lymphocytes by modifying Th1 type immune response and local infiltration of Th17 cells* in vivo*. This study might provide a clue for developing a useful strategic alternative for clinical biotherapy for tumor patients.

## Figures and Tables

**Table 1:** The clinical pathological characters of the patients with lung cancer.

Clinical pathological parameter	*n*
sex	
Male	15
Female	11
Age (years)	18–72
Tumor stage	
T1/2	12
T3/4	14
Nodal status	
N0/1	15
N2/3	11
Histological type	
NSCLC	21
SCLC	5

(1) Lymph nodal metastasis is according to pathological diagnosis and clinical palpation;

(2) clinical stage is according to TNM stage.
